# Surgical Treatment of Vulvar HSIL: Adjuvant HPV Vaccine Reduces Recurrent Disease

**DOI:** 10.3390/vaccines9020083

**Published:** 2021-01-25

**Authors:** Alessandro Ghelardi, Roberto Marrai, Giorgio Bogani, Francesco Sopracordevole, Paola Bay, Arianna Tonetti, Stefania Lombardi, Gloria Bertacca, Elmar A. Joura

**Affiliations:** 1 Azienda USL Toscana Nord-Ovest, UOC Ostetricia e Ginecologia, Ospedale Apuane, Via Enrico Mattei, 21, 54100 Massa, Italy; roberto.marrai@uslnordovest.toscana.it (R.M.); paolabay@aliceposta.it (P.B.); arianna.tonetti@uslnordovest.toscana.it (A.T.); 2 Gynecological Oncology Unit, Fondazione IRCCS Istituto Nazionale dei Tumori di Milano, Via Giacomo Venezian, 1, 20133 Milano, Italy; giorgio.bogani@istitutotumori.mi.it; 3 Centro di Riferimento Oncologico, National Cancer Institute, Gynecological Oncology Unit, Via Franco Gallini, 2, 33081 Aviano, Italy; fsopracordevole@cro.it; 4 Azienda USL Toscana Nord Ovest, SSD Analisi Chimico Cliniche ed Immuno Allergologia, Ospedale Apuane, Via Enrico Mattei, 21, 54100 Massa, Italy; stefania.lombardi@uslnordovest.toscana.it (S.L.); gloria.bertacca@uslnordovest.toscana.it (G.B.); 5 Department of Gynecology and Obstetrics, Comprehensive Cancer Center, Medical University of Vienna, Austria Währinger Gürtel 18-20, 1090 Vienna, Austria; elmar.joura@meduniwien.ac.at

**Keywords:** HPV vaccination, adjuvant HPV vaccination, vulvar HSIL, vulvar HSIL treatment

## Abstract

Data suggest that adjuvant human papillomavirus (HPV)-vaccination in women treated for cervical HPV diseases reduces recurrent disease. This study investigates adjuvant HPV-vaccination and the rate of recurrence in women undergoing surgery for vulvar high-grade squamous intraepithelial lesions (HSIL). From January 2013 to April 2020, we enrolled 149 women in a prospective case-control study. The control group (NV-group) was treated by standard surgery alone, while the study group received adjuvant vaccination soon after surgery (V-group). A follow-up was performed by vulvoscopy and HPV test. Statistical analysis was performed by Fisher’s exact test. HSIL recurrence was observed in 24/76 (32%) patients in NV-group and in 8/42 patients (19%) of the vaccinated group. By analysing the recurrence rate related to the incident and reactivated latent HPV infection, we found a significant difference between (17/76) 22.3% in NV-group and (2/42) 4.8% in V-group (*p* = 0.01). A reduction of 78.5% in incident/reactivated HPV infections was demonstrated. Data results add to the current knowledge about the mechanism of post-surgical adjuvant HPV vaccination. Our prospective study is the first to document the vaccine clinical effectiveness in preventing “reactivation” of latent HPV infections. Quadrivalent HPV vaccine administered after the surgical treatment for vulvar HSIL appears to be useful in preventing recurrent disease.

## 1. Introduction

High-risk human papillomavirus (HPV) types cause approximately 5% of all cancers worldwide [1]. In the United States, high-risk HPV types cause approximately 3% of all cancer cases among women and 2% of all cancer cases among men [2]. Virtually all cases of cervical cancer are caused by HPV, and just two HPV types, 16 and 18, are responsible for about 70% of all cases. Persistent human papillomavirus infection with oncogenic types has also been linked to 70% of vulvar cancers [3]. In Italy, it is estimated that there are about 2600 new cases/year of cancer of the cervix and 390 cases/year of vaginal-vulvar tumours [4]. A recent article shows the average lifetime probability of acquiring HPV among those with at least one opposite-sex partner to be 84.6% (range, 53.6–95.0%) for women and 91.3% (range, 69.5–97.7%) for men [5]. In other words, human papillomaviruses are the most widespread and common sexually transmitted infections worldwide [6]. According to these clinical findings, the only efficacious strategy, in order to reduce HPV infections is primary HPV vaccination.

In 2006 HPV vaccination was licensed for primary prevention of HPV related diseases in young females [7]. Although the peak incidence of human papillomavirus infection occurs in most populations within 5–10 years of first sexual experience, all women remain at risk for acquisition of HPV infections [8,9]. For this reason, a recent study investigated adult women vaccination, showing that HPV quadrivalent vaccination is efficacious in women aged 24–45 years not infected with the relevant HPV types at enrolment [10]. The quadrivalent HPV vaccine was the first prophylactic HPV vaccine, and it was shown to be highly effective in preventing cervical and vulvovaginal pre-neoplastic lesions in young women aged 16–26 [11]. Later, the quadrivalent HPV vaccine demonstrated efficacy against HPV infection and disease in women up to 45 years [10,12].

Persistent HPV infection and high-grade squamous intraepithelial lesions (HSIL) are presumed to lead to HPV-related vulvar cancer, as persistent cervical HPV infection can lead to cervical cancer. The protective role of the HPV vaccine in preventing vulvar premalignant lesions (Vulvar HSIL according to clinical and molecular classification of vulvar squamous pre-cancers [13] or VIN (vulvar intraepithelial neoplasia classification) [14], was well documented. In 2004, VIN were classified into two groups by the International Society for the Study of Vulvar Disease (ISSVD): Usual-type VIN (uVIN) and differentiated type (dVIN). Differentiated type is commonly related to vulvar dermatoses, typically lichen sclerosus and affects older women, while uVIN is related to HPV infection and associated with carcinogenic subtypes of human papillomavirus. The worldwide incidence of vulvar pre-cancers in young women has been increasing [15,16,17,18,19], moving from 1.2/100.00 to 2.1/100.000 women over recent decades. Vulvar HSIL is usually caused by HPV 16 and is a precursor of HPV related vulvar cancer which is also predominately caused by HPV 16 and 33 [20,21]. This is in contrast to dVIN, which is a precursor of HPV negative vulvar cancers [22]. Based on current knowledge HPV vaccination is not believed to alter the course of prevalent lower genital tract diseases, although data coming from clinical trials suggested a statistically significant impact in reducing post-surgical recurrent disease Although HPV vaccine is not therapeutic in patients with prevalent HPV infection or disease, recent data suggest that vaccination, in women who underwent surgical therapy, has a significant impact on disease recurrence [23]. 

Based on the aforementioned knowledge, we decided to evaluate the possible impact of receiving a post-surgical HPV-vaccination in women treated for vulvar HSIL. 

In January 2013 Azienda USL-1 Massa e Carrara approved a clinical project called “SPER.AN.Z.A.” (Sperimentazione Anti HPV Zona Apuana, an Italian acronym for HPV vaccination clinic for adult women). Patients are referred to the clinic by 11 local hospitals and private gynaecologists for adjuvant vaccination, and all referred women have been included in the project. The patient population of the USL Toscana nord ovest area is about 1,000,000 women. Previously we reported a reduction of subsequent cervical disease after conisation of 81.2% [24].

The aim of this study was to investigate possible implications of receiving adjuvant HPV vaccination, in individuals surgically treated for vulvar H-SIL, evaluating the *clinical effectiveness* of the human papillomavirus 4-valent vaccine in preventing disease relapse after surgery. 

## 2. Methods

**Figure 1:**
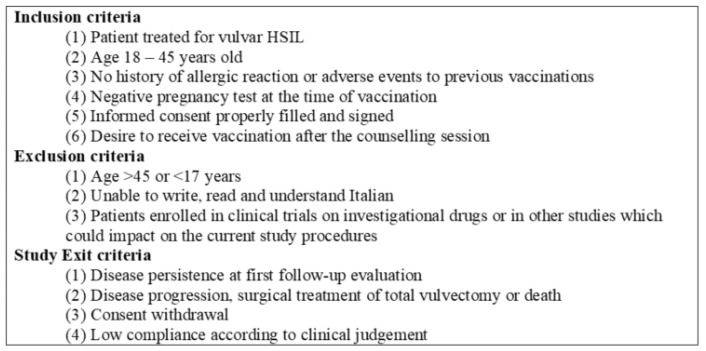
Enrolment criteria.

**Figure 2:**
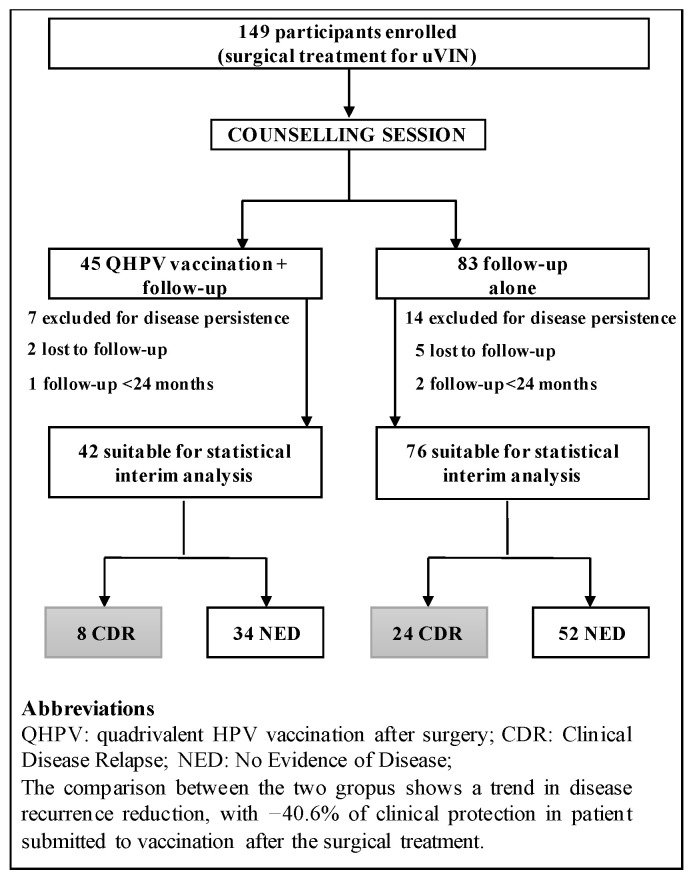
Study flow chart with participant’s disposition. HSIL, high-grade squamous intraepithelial lesions.

Participation in the study was offered to all women undergoing surgical treatment for vulvar HSIL, between January 2013 and April 2020, according to the inclusion/exclusion criteria (Figure 1). All the patients treated for vulvar HSIL were invited to a counselling session (see Figure 2), which was performed in the SPERANZA vaccination clinic. 

Women were enrolled in this prospective case-control study, regardless of their baseline HPV DNA status. The main outcome was to evaluate the incidence of subsequent HPV-related vulvar HSIL during the follow-up period between the two groups. 

The study was conducted according to the ethical standards of the Declaration of Helsinki. Written informed consent was obtained from all the participants; the clinical management was in accordance with Good Clinical Practice, and was approved by the ATNO area. The following pre- and post-surgical procedures are standardised.

### 2.1. Pre-Surgical Clinical Evaluation

Vulvoscopy, punch biopsy for histological assessment and HPV test were performed during the pre-surgical evaluation; patients with a vulvar HSIL diagnosis were referred to surgical treatment. 

HPV test was collected by vulvar scraping, and a commercial kit (HPV sign; Diatech, Jesi, Italy) was used to detect the presence of virus by real-time PCR of L1 region of HPV and the human beta-globin gene as the internal control. The reverse specific primers for HPV detection were biotinilated. The thermal profile performed on a RotorGeneTM 6000 instrument (Corbett, New South Wales, Australia) was 95 °C for 3 min, 50 cycles at 95 °C for 0.5 min; 44 °C for 0.5 min; 72 °C for 0.5 min; followed by a final melting from 72 °C to 95 °C with 1 °C increment at each step. The obtained melting curve was analysed in order to assign the positivity of the sample.

The byotinilated HPV positive PCR products were subjected to pyrosequencing using a commercial kit (HPV sign; Diatech, Jesi, Italy). After some steps of purification from the reaction mixture with the PyroMark Q96 ID system (Qiagen, Hilden, Germany) according to themanufacturer’s instructions, the DNA was denaturated by heating and added to sequence HPV primers. The sequencing reaction was performed in the presence of substrate and enzyme by dispensation of dATPαS, dCTP, dGTP and dTTP for 15 cycles on PyroMark Q96 ID system (Qiagen, Hilden, Germany). The specific HPV sequences were 30 bp length and were compared with those contained in the library of IdentiFireTM software that assigned the genotype by a score of percentage of identity.

### 2.2. Surgical Treatment

The surgical treatment was colposcopy guided and generally performed under local anaesthesia (using 2% Carbocaine). Most cases underwent electrosurgical wide local excision and/or laser vaporisation; small lesions in periclitoral area or lesion with minimal residual vulvar disease post-biopsy underwent laser vaporisation only. A solid-state erbium in yttrium aluminium-garnet crystal (Er:YAG) laser (Fotona Smooth™ XS, Fotona, Ljubljana, Slovenia) with a wavelength of 2940 nm, set to ablative mode was utilised. 

### 2.3. Counselling Session

A first clinical evaluation at the vaccination clinic was scheduled for all the patients with histologic confirmed vulvar HSIL within 30 days after surgery. All women in line with inclusion and exclusion criteria (Figure 1) were informed of the opportunity to participate in a counselling session allowing women to discuss their problems and any difficulties about HPV diseases in a safe, confidential environment. Counselling was free of charge. 

### 2.4. Enrolment Procedure, Variables Collected

After the counselling session, participation in the study was offered to all patients treated for vulvar HSIL (Figure 2). Variables related to recurrence risk were also collected for each patient, such as multifocality of the lesion, periclitoral involvement, smoking status, age of the patient. 

#### 2.4.1. Case Group (V-Group)

Patients satisfying enrolment criteria and desiring to receive the vaccine, were included in the study as “vaccinated and follow-up” arm. All “vaccinated” patients received HPV quadrivalent vaccine (types 6,11,16,18 L1 virus-like particle vaccine Gardasil, Merck, Whitehouse Station, NJ, USA). Patients received vaccination with the first dose after counselling and the remaining two doses 2 and 6 months later. The vaccine was offered to a reduced price.

#### 2.4.2. Control Group (NV-Group)

Women fitting enrolment criteria, once filled out the informed consent and not desiring vaccination, were enrolled in the “follow-up only” arm, as the control group (NV-group). 

### 2.5. Post-Surgical Follow-Up

A post-surgical evaluation was performed with the vulvar HPV test and vulvoscopy; the first follow-up evaluation was scheduled every three months in the first year after the surgery, then participants were evaluated annually until the seventh year post-treatment.

Any vulvar area suspected of disease persistence or recurrence was investigated by punch biopsy. Recurrences were treated with re-excision of the area and confirmed by histological diagnosis, while vulvectomy associated with inguinal-femoral lymphadenectomy was performed in case of invasive vulvar carcinoma. Persistent vulvar HSIL at first clinical evaluation was a study exit criteria Data were collected in a regional database. A vaccination certificate was issued to the patient. Adverse events were reported to offices in charge, as per clinical practice. Only women followed for at least 24 months were included in the analysis.

### 2.6. Definition of Clinical Disease Relapse, Prevalent and Incident Recurrence

Clinical disease relapse (CDR) was defined as a clinical disease recurrence, histologically confirmed, of HSIL during the seven years follow-up period. 

**Figure 3:**
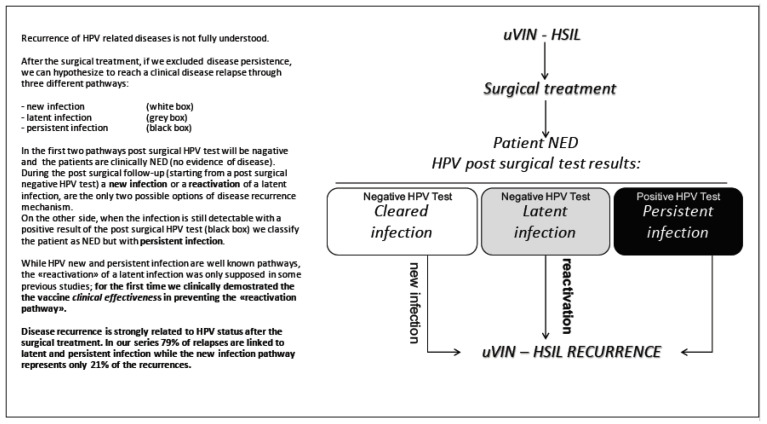
Schematic model of human papillomavirus (HPV) related disease recurrence.

In order to understand the mechanism of relapse, we divided the recurrences into three categories, based on the pathway of the HPV infection status after the surgical treatment. The clinical findings of this evaluation are reported in Figure 3 (schematic model of HPV related disease recurrence).

With the term of “Persistent Recurrence” (PR), we defined a subgroup of patients, who relapsed with a histologically confirmed HSIL related to the same HPV type found in the first surgical treatment, during the follow-up period.

With the term of “Incident Recurrence” (IR), we define a relapse associated with an HPV “de novo” infection (due to an HPV type that was not present at the time of the first diagnosis), while “reactivation” was called an infection caused by the same HPV type found at first surgery.

While the CDR absolute number includes both persistent and incident recurrences together, the subgroup analysis (PR/IR) of our model of recurrence is able to determine the exact role of persistent and incident infections.

## 3. Data Analysis

CDR, PR, IR, variables associated with recurrence risk in both groups were calculated. Women finally lost to follow-up remained in the analysis for the period we could follow them, and then they were censored. A *p* value of <0.05 was considered statistically significant. The Fisher’s exact test was used to assess the significance of differences between the two groups.

## 4. Results

### 4.1. Study Population

During the study period, a total of 149 patients that underwent surgery for vulvar HSIL were enrolled (Figure 2) for disease persistence at the first follow-up visit, two were lost to follow-up, and one had a follow-up period shorter than 24 months. In the NV-group 14 patients were excluded for disease persistence at the first follow-up visit, five were lost to follow-up, and two has limited follow-up time (Figure 2).

For the 118 women included in the analysis, variables related to recurrence risk: Age, multifocality, periclitoral area involvement and smoke status, were evaluated. No factor was associated with the risk of developing recurrence at univariate analysis (no measurable factor was associated (*p* < 0.2)/correlate (*p* < 0.05) with the aforementioned recurrence risk factors). Therefore, a multivariate analysis was not built (Table 1). HPV types of distribution identified at enrolment are shown in Table 2. At data cut off, the median follow-up was close to five years (58 months, range 24–84 months).

### 4.2. HPV Natural History after the Surgical Treatment 

Three months after the surgery, in the non-vaccinated group, 26/76 (34.2%) women were HPV positive with an HPV infection clearance rate of 65.8%, while the vaccinated group (V-group) showed a positive HPV test in 11/42 (26.2%), with an HPV infection clearance rate of 73.8%. 

There was no statistically significant difference between the two groups: Chi-square value *p* = 0.41. At recurrence, the most frequent type of HPV in NV group was HPV 16 (71%) followed by HPV 33 (21%) and HPV 18 and 51 (8% each) in V group again HPV 16 was the most frequent type (50%) followed by HPV 33 (25%) (Table 3).

### 4.3. Clinical Effectiveness of Quadrivalent HPV Vaccine after Surgical Treatment

Data of 118 women with 24-months or more of follow up were analysed for CDR. 

CDR HSIL recurrence was observed in 24/76 (32%) patients in NV-group; vaccinated group developed HSIL recurrence in 8/42 patients (19%). CDR analysis shows a trend in disease reduction (*p* = 0.19), with −40.6% of clinical relapses in V-group. 

### 4.4. Recurrent Disease: Pathways Analysis

**Figure 4:**
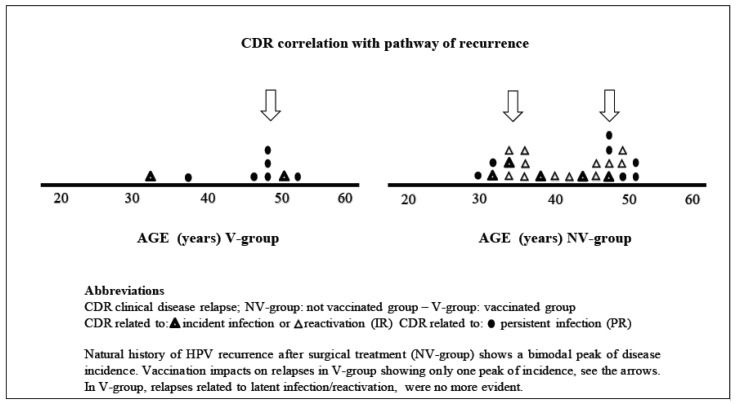
CDR subgroup analysis << Persistent/Incident recurrence >>(PR/IR).

Analysing the different pathways of recurrent disease (Figure 4) we observed that the number of relapses, due to “new infection” were 2/8 in V-group vs. 5/24 of NV-group (*p* = 0.63). 

In NV-group we observed 12/24 women with recurrent disease via “reactivation pathways”, while in V-group showed no disease relapse, due to reactivation; difference between the two groups was statistically significant (*p* = 0.01).

Globally the pathways related to “persistent infection vs. incident/reactivated the previous infection”, the difference between the two groups was statistically significant, with a recurrence rate of 22.3% in the NV-group and 4.8% in the V-group, with a *p*-value = 0.01 (by Pearson’s chi squared test) and a *clinical effectiveness of −78.5% in reducing HPV relapse.*

### 4.5. Age Distribution Analysis at the Time of the CDR

Age distribution at the time of recurrent disease was analysed in Figure 4. The graph with natural HPV history after the treatment (NV-group) shows a bimodal peak incidence of recurrence (the first around 35 years and the second in perimenopausal age), while in the V-group the first peak of recurrence was no more evident. 

### 4.6. Adverse Events of Vaccination, Safety

Vaccination was well-tolerated by all the patients; no major adverse events were reported. Common minor adverse events (muscle and joint pain, headache, fever, pain, redness or swelling in the arm where the shot was given) were well-tolerated too, with complete resolution of the symptoms within two weeks. No discontinuations in the study, due to AE were observed.

## 5. Discussion

Vulvar HSIL is considered the precursor lesion for vulvar squamous cell carcinoma. Early diagnosis and adequate treatment are the only strategies for secondary prevention currently available. Analysing surgical treatments, a Cochrane review [25] confirm that surgical excision and laser vaporisation may be equally effective treatments for HSIL, and about half of women will experience HSIL recurrence after either approach. This knowledge underlines not only the need for a long-term follow-up after the surgery, but also how morbidity and the high recurrence rates associated with surgical cares make new perspectives desirable. 

In order to reduce disease relapse, adjuvant vaccination in HPV related disease is an upcoming issue [26,27,28,29,30,31,32,33,34,35,36]; this clinical topic is analysed by a very recent review investigating about the use of prophylactic vaccines in patients treated for HPV related pathologies [37]. 

The interest about the potential role of HPV adjuvant vaccination is confirmed by the “VIVA trial” [38], an ongoing RCT investigating about the possibility of post-surgical HPV vaccination in order to reduce vulvar and anal HSIL recurrence. The role of HPV vaccine in patients with prevalent infection is still controversial—a very recent article analysed this open question [39]. The study investigated the hypothesis that HPV vaccination administered in patients with low-grade cytology could prospectively alter HPV-related biomarkers. The main purpose of the study was to examine the evolution of documented single HR HPV infections in individuals who subsequently received the HPV vaccine in comparison to the controls who did not. In this prospective pilot study, a total of 309 women were included to the final analysis, subdivided into 20 separate subgroups in total based on the HPV subtype at the initial assessment (HPV DNA genotyping).

HPV vaccination reduced in a statistically significant manner (*p* < 0.05) HPV DNA positivity rates for genotypes 16, 18 and 31, RR = 1.6 (95% CI: 1.1 to 2.3), RR = 1.7 (95% CI: 1.1 to 2.8), and RR = 1.8 (95% CI: 1.0 to 2.9), in women who only tested DNA-positive for HPV16, 18 and 31 genotypes, respectively, prior to vaccination, demonstrating an earlier clearance of HPV infection in comparison with the patients included in the non-vaccination cohort. 

Numerous adjuvant vaccination studies [26,27,28,29,30,31,32,33,34,35,36] are available, demonstrating that HPV vaccine can reduce clinical disease relapse after the surgical treatment, but the mechanism is not fully understood. 

Our data are also comparable to a very recent article published by Sand et al. [40] who found a trend in disease reduction in patients submitted to adjuvant vaccination 0–3 months after surgical treatment (in our series global recurrence rate was 32% vs. 19% in NV group vs. V-group, respectively, *p* value *=* 0.19, with −40.6% of clinical relapses in V-group). Similar data were also published in a post-hoc analysis of Joura et al. [23] in which vaccination reduce by 30.1%. The recurrent of high-grade VIN 60 days or more after surgery irrespective of HPV type.

Vulvar HSIL recurrence rate after excision ranges from 20% to 40% [41]. This wide range is probably related to the definition of “clinical disease recurrence” (CDR), where there is no global consensus on relapse terminology. Literature data show no distinction from repeated surgery, due to locally persistent disease, de novo HPV infection or latent and persistent post-surgical viral status.

Clinical data coming from the “SPERANZA project” about viral immunology allowed us to design a model of the pathways of disease recurrence (Figure 3). While the “global recurrence” absolute number includes both persistent and incident recurrences, we conducted a subgroup analysis “persistent vs. incident recurrence (PR/IR)” (Figure 4), in order to determine the exact role of persistent and incident infections.

Analysing the pathways of recurrence in our series we observed that vaccination is able to influence the natural history of HPV infection after the surgery: We found a minor difference between the groups in recurrence, due to “de novo” infection (2/8 V-group vs. 5/24 NV-group—*p* = 0.63).

Focusing on the “reactivation” the difference between the two groups is statistically significant. In the V-group not a single reactivation of a latent infection was observed, while in the NV-group a reactivation of a latent infection happened in half (12/24) of the women (*p* = 0.01). For the first time, we demonstrated that the mechanism of recurrence reduction is not only, due to secondary prevention of “de novo infections” (as already hypothesised in previous studies [23,42]), but also based on prevention of “HPV reactivation” in vulvar disease (Figure 3).

In Figure 4, we analysed how HPV natural history of recurrence after surgical treatment (NV-group) shows a bimodal peak of disease incidence. Vaccination strongly impacts relapse in V-group, showing only one peak of incidence, as in V-group, relapses related to latent infection reactivation, were no more evident. Although HPV vaccination is not thought to alter the course of disease in women with prevalent type-specific infections, we clinically demonstrate that adjuvant vaccine can change the post-surgical natural history of HPV infections, by secondary prevention. SPERANZA project was conducted using HPV quadrivalent vaccine, while nowadays nonavalent vaccination is currently available standard, this could lead to an even higher efficacy in reducing the incidence of HSIL recurrence, since a substantial part of recurrent disease was related to HPV 33.

Some previous studies [43,44,45,46,47,48,49,50,51] have shown that the application of topical imiquimod 5% is an effective option for the treatment of vulvar HSIL. In a recent Cochrane review [28] topical imiquimod, after five to six months of treatment, showed a complete response in up to 58% of patients. Main issues with this approach are time-related (up to 12–20 weeks of therapy), difficulties, due to self-administration, adverse events (common: Headache, fatigue, vulvar pain) and in case of failure (due to residual disease) the need of surgery. For those reasons, our study design with a “two steps” approach, surgery followed by vaccination, could offer a new clinical perspective in terms of fast results and a lower rate of recurrent disease.

Our results add to the current knowledge on the mechanism of adjuvant vaccination in women treated for HPV related diseases, however, future studies are mandatory to confirm the findings.

Strengths of the study include active follow-up of participants over seven years after the surgical treatment, with a rigorous assessment of procedures and endpoints. It is important to note that all participants were intensively tested for HPV infections, before and after surgery, which gives the opportunity to draw a natural history model of post-surgical HPV infections, understanding the pathways of disease recurrence. Limitations of our study include the modest absolute number of participants, due to the fact that vulvar HSIL represents a rare condition; moreover, even if this is a non-randomised trial, the comparison of secondary risk factors for disease recurrence between the two groups was not statistically significant, mitigating this important limitation.

## 6. Conclusions

The quadrivalent HPV-vaccine, administered after surgical treatment for vulvar HSIL, may be useful in preventing recurrence of the disease. The HPV vaccination is safe, efficacious and well-tolerated in women with vulvar HSIL. These results of our prospective, not randomised trial, adds to the current knowledge about the mechanism of adjuvant vaccination. A large multicentre randomised trial would finally confirm these findings.

## Figures and Tables

**Table 1:** Distribution of study subjects according to selected characteristics and treatment group.

Characteristic	NV-Group (76 pts)		V-Group (42 pts)		Chi Square Test Value (*p* Value)
	No.	(%)	No.	(%)	
**Multifocality/unifocality**	24/52	31.5/68.5	9/33	21.4/78.6	0.28
**Periclitoral area involvement/other areas**	10/62	13.1/86.9	7/35	16.6/83.4	0.59
**smokers**	35	46	23	54.7	0.44
**Median age**	40.6		41.2		

**Abbreviations:** NV-group, not vaccinated group; V-group, patients submitted to quadrivalent HPV vaccine post-surgery.

**Table 2:** HPV types of distribution identified at enrolment.

HPV Type	NV-Group (83 pts)		V-Group (45 pts)	
	No. of Patients	%	No. of Patients	%
High-risk HPV				
16	53	64	28	62
18	9	11	3	7
33	6	7	9	20
45	1	1		
51	5	6	3	4
56	2	2		
58	1	1		
68			1	2
Low-risk HPV				
11	13	16	9	20
6	6	7	4	9
undetectable	3	4		

**Abbreviations:** CDR, clinical disease relapse. NV-group, not vaccinated group; V-group, patients submitted to quadrivalent HPV vaccine post-surgery.

**Table 3:** HPV types of distribution identified at clinical disease recurrence.

HPV Type	NV-Group (24 pts)		V-Group (8 pts)	
	No. of CDR	%	No. of CDR	%
16	17	71	4	50
18	2	8		
33	5	21	2	25
51	2	8		
56	1	4	1	12.5
58	1	4	1	12.5

**Abbreviations:** CDR, clinical disease relapse. NV-group, not vaccinated group; V-group, patients submitted to quadrivalent HPV vaccine post-surgery.

## Data Availability

No new data were created or analyzed in this study. Data sharing is not applicable to this article.
